# MIRU-profiler: a rapid tool for determination of 24-loci MIRU-VNTR profiles from assembled genomes of *Mycobacterium tuberculosis*

**DOI:** 10.7717/peerj.5090

**Published:** 2018-07-11

**Authors:** Rahim Rajwani, Sheeba Shehzad, Gilman Kit Hang Siu

**Affiliations:** Department of Health Technology and Informatics, Hong Kong Polytechnic University, Hong Kong, China

**Keywords:** *Mycobacterium tuberculosis*, MIRU-VNTR, TB transmission, Whole genome sequencing

## Abstract

**Background:**

Tuberculosis (TB) resulted in an estimated 1.7 million deaths in the year 2016. The disease is caused by the members of *Mycobacterium tuberculosis complex*, which includes *Mycobacterium tuberculosis, Mycobacterium bovis* and other closely related TB causing organisms. In order to understand the epidemiological dynamics of TB, national TB control programs often conduct standardized genotyping at 24 Mycobacterial-Interspersed-Repetitive-Units (MIRU)-Variable-Number-of-Tandem-Repeats (VNTR) loci. With the advent of next generation sequencing technology, whole-genome sequencing (WGS) has been widely used for studying TB transmission. However, an open-source software that can connect WGS and MIRU-VNTR typing is currently unavailable, which hinders interlaboratory communication. In this manuscript, we introduce the MIRU-profiler program which could be used for prediction of MIRU-VNTR profile from WGS of *M. tuberculosis.*

**Implementation:**

The MIRU-profiler is implemented in shell scripting language and depends on EMBOSS software. The in-silico workflow of MIRU-profiler is similar to those described in the laboratory manuals for genotyping *M. tuberculosis*. Given an input genome sequence, the MIRU-profiler computes alleles at the standard 24-loci based on in-silico PCR amplicon lengths. The final output is a tab-delimited text file detailing the 24-loci MIRU-VNTR pattern of the input sequence.

**Validation:**

The MIRU-profiler was validated on four datasets: complete genomes from NCBI-GenBank (*n* = 11), complete genomes for locally isolated strains sequenced using PacBio (*n* = 4), complete genomes for BCG vaccine strains (*n* = 2) and draft genomes based on 250 bp paired-end Illumina reads (*n* = 106).

**Results:**

The digital MIRU-VNTR results were identical to the experimental genotyping results for complete genomes of locally isolated strains, BCG vaccine strains and five out of 11 genomes from the NCBI-GenBank. For draft genomes based on short Illumina reads, 21 out of 24 loci were inferred with a high accuracy, while a number of inaccuracies were recorded for three specific loci (ETRA, QUB11b and QUB26). One of the unique features of the MIRU-profiler was its ability to process multiple genomes in a batch. This feature was tested on all complete *M. tuberculosis* genome (*n* = 157), for which results were successfully obtained in approximately 14 min.

**Conclusion:**

The MIRU-profiler is a rapid tool for inference of digital MIRU-VNTR profile from the assembled genome sequences. The tool can accurately infer repeat numbers at the standard 24 or 21/24 MIRU-VNTR loci from the complete or draft genomes respectively. Thus, the tool is expected to bridge the communication gap between the laboratories using WGS and those using the conventional MIRU-VNTR typing.

## Introduction

Tuberculosis (TB) is an infectious disease responsible for an estimated 1.7 million deaths worldwide in the year 2016 alone (World Health Organization, 2017). In a global epidemic situation, genotyping of *Mycobacterium tuberculosis complex* strains (the causative agent of TB) is crucial for TB control particularly via epidemiological surveillance and monitoring TB-transmission.

The gold-standard method for genotyping *M. tuberculosis* has changed over time. In the 1990s, IS*6110* restriction-fragment-length-polymorphism (RFLP) was recognized as the gold-standard for strain-discrimination and genotyping of *M. tuberculosis* (Otal et al., 1991). However, the laboratory procedure for IS*6110*-RFLP is time and labour-intensive. It requires large amount of pure DNA, which consequently requires weeks of bacterial cultivation. In 2001, mycobacterial-interspersed-repetitive-units (MIRU)-variable-number-of-tandem-repeats (VNTR) typing was developed, which is a polymerase-chain-reaction (PCR)-based method that could be performed on the crude DNA-extracts (Mazars et al., 2001).

The MIRU-VNTR typing examines polymorphisms in the number of repeat units at the selected VNTR-loci. The VNTRs are human minisatellite-like markers, that are composed of 50–100 bp repeat units, and are present at divergent locations on the genome. The laboratory procedure involves a PCR reaction with primers flanking the VNTR-loci and sizing of the PCR-amplicon subsequently. Since the length of the repeat unit is known, the number of copies of the repeat unit could be computed based on the PCR amplicon-size. Based on an analysis of over 800 clinical isolates from 28 countries, the discriminatory power for a set of 24 MIRU-VNTR loci was comparable to the previous gold standard genotyping method (i.e., IS*6110*-RFLP) (Supply et al., 2006). This standardization was performed by an international consortium, comprising of 11 European and American laboratories, which subsequently led to the establishment of 24-loci MIRU-VNTR typing as the new gold standard for genotyping *M. tuberculosis*, particularly for the routine epidemiological and phylogenetic analysis. The 24-loci MIRU-VNTR typing data is collected for all *M. tuberculosis* isolates by the public health laboratories in the Nordic countries (Denmark, Finland, Sweden), the Netherlands and the United Kingdom. Moreover, 21 European-Union/European-economic-area member-states also submit the genotyping data of at least 80% of the drug-resistant isolates to the European Centre for Disease Prevention and Control (ECDC) (ECDC, 2017). Since 2009, the United States (US)-CDC has implemented a nationwide program for 24-loci MIRU-VNTR typing of all *M. tuberculosis* isolates and has genotyped over 120,000 clinical strains already (CDC, 2008).

In the recent years, the whole-genome-sequencing (WGS) of *M. tuberculosis* has emerged as a molecular epidemiological tool. New reports have been published describing the applications of WGS for TB outbreak detection and investigating the direction of TB transmission (Folkvardsen et al., 2017; Satta et al., 2017; Shah et al., 2017). The WGS has the potential to distinguish between two strains differing by only a single nucleotide. It also reveals additional information related to drug-resistance and virulence. However, several obstacles yet remain before WGS could replace the current gold standard for *M. tuberculosis* genotyping (i.e., MIRU-VNTR typing). Foremost, defining a transmission cluster based on WGS is challenging. The preliminary studies have assigned *M. tuberculosis* strains to the same transmission-cluster if the genetic distance between them do not exceed five to twelve single nucleotide polymorphisms (SNPs) (Walker et al., 2013). However, this cut-off is expected to vary among human populations, sequencing platforms, bioinformatics pipelines, and phylogenetic lineage of the *M. tuberculosis*. A core-genome based multi-locus-sequence-typing is suggested as an alternative to the SNP-based clustering, however, a prospective evaluation of this approach on a large sample size is yet to be performed (Kohl et al., 2014). In brief, while WGS provides the highest resolution for epidemiological investigations, extensive standardization is required before its introduction into the clinical settings.

So far, fewer isolates have WGS data than MIRU-VNTR typing results. Over the past decade, MIRU-VNTR data has been collected for millions of isolates and are stored in public databases along with the detailed clinical and epidemiological information. As WGS is becoming more common for surveillance study, a bioinformatics tool is required to infer the MIRU-VNTR profiles from the genome sequences. This will facilitate an inter-laboratory communication between countries that have already implemented WGS and countries that use conventional MIRU-VNTR genotyping method.

In this report, an open-source bioinformatics tool “MIRU-profiler” is introduced, which predicts the standard 24-loci MIRU-VNTR profiles from multiple genome assemblies of *M. tuberculosis* in a single batch on any standard computer. The MIRU-profiler will facilitate effective linkage of genome-sequenced strains with MIRU-VNTR genotyped isolates, which has become critically important in the transitionary period of WGS as a routine epidemiological tool and continuous threats of TB outbreaks worldwide.

## Materials and Methods

### Construction and specification of the program

**Figure 1:**
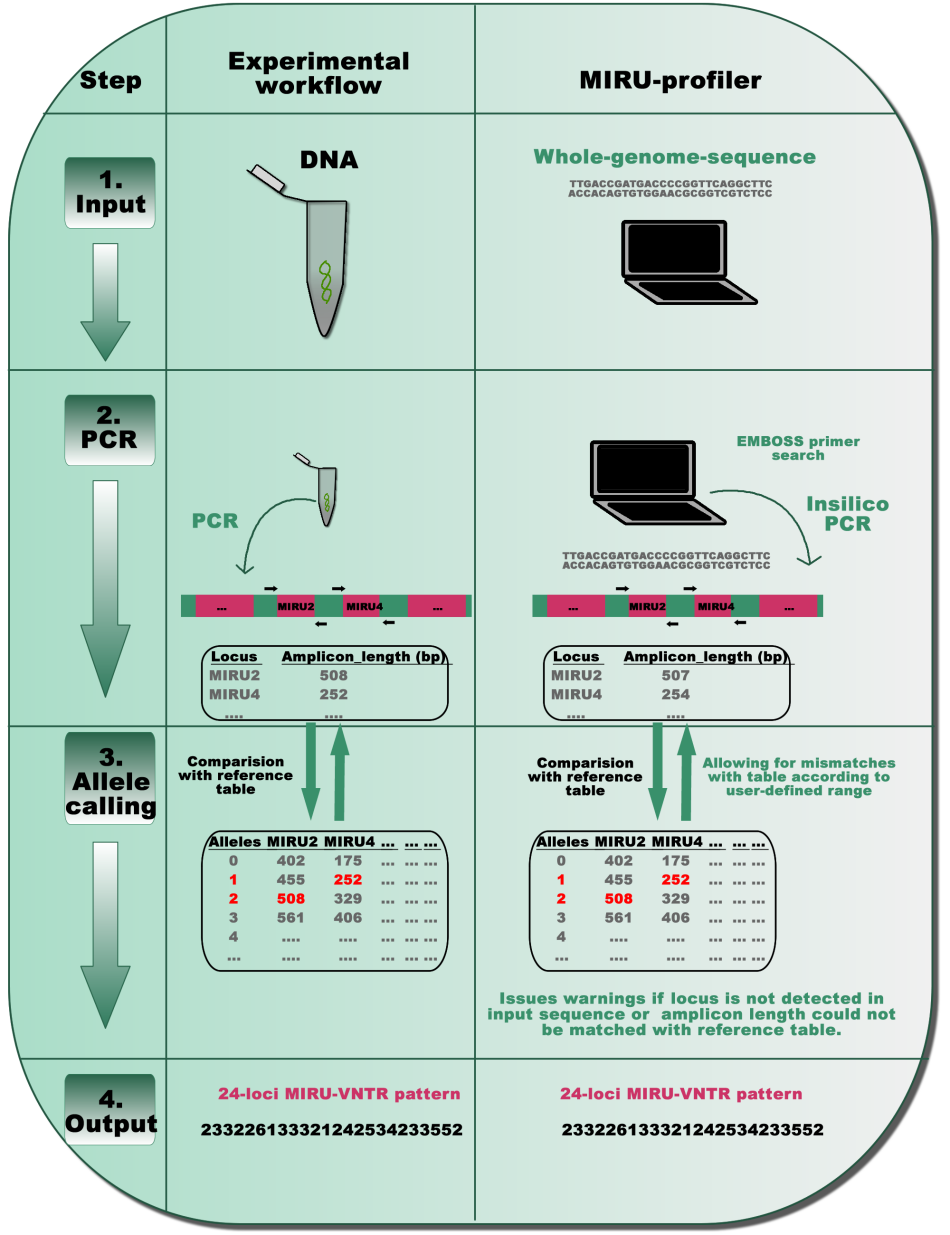
A schematic representation of the MIRU-profiler workflow in comparison with the experimental protocol for the standardized genotyping of *M. tuberculosis* using 24 MIRU-VNTR loci. The similarity between the MIRU-profiler and experimental workflow is illustrated at four stages: input, PCR, allele-calling and output. By simulating the experimental workflow in-silico, the MIRU-profiler is designed to infer the 24-loci MIRU-VNTR pattern from whole-genome-sequences of *M. tuberculosis*. As indicated at the stage 4 output, the 24-digit numeric pattern inferred by the MIRU-profiler might be identical and comparable to those obtained by a wet-laboratory experiment.

The MIRU-profiler is written in shell scripting language and is useful for digital genotyping *M. tuberculosis* strains based on standard 24-loci MIRU-VNTR. The program input is a FASTA file of whole-genome-sequence assembled using reads of 250 bp or longer. The MIRU-VNTR loci are not accurately assembled in genome assemblies with very short reads (for example 150 bp), therefore, assemblies based on them are not recommended as an input to the program (Table S1). The final output is the number of repeats at the 24 MIRU-VNTR loci in a tab-delimited text file for easy manipulation in spreadsheet editors (Microsoft Excel or LibreOffice Calc). The entire code is embedded in a loop which enables the program to accept multiple files as input and process them in a batch mode. Detailed installation and usage instructions are provided on the project homepage: https://github.com/rahimrajwani/MIRU-profiler. The in-silico workflow of the MIRU-profiler is similar to those recommended in previously published laboratory manuals (Supply, 2005) (Fig. 1).

First, standard PCR primers (Supply, 2005; Supply et al., 2006) are used to compute amplicon lengths for all 24 MIRU-VNTR loci in a given genome sequence using the EMBOSS primer search program (Rice, Longden & Bleasby, 2000). In order to account for the sequencing errors or polymorphisms within the primer sequences, the MIRU-profiler allows up to three mismatches (∼15%) within 20–24 nucleotides long primer sequences. In cases where multiple amplicons are detected, the amplicon resulting from the least number of mismatches within the primers, is further analyzed in the subsequent step. If an amplicon corresponding to a particular locus is not detected at all (either due to no hits for the primer, genomic deletion or absence of a corresponding contig), the program flags the locus as “NOT_DETECTED”.

Second, the number of repeats at the VNTR loci is computed based on amplicon-lengths as per the routinely used reference allele-calling table (Supply, 2005). At this step, the MIRU-profiler allows a user defined discrepancy range (in base-pairs) between the amplicon length in input sequence and the allele-calling table. If the amplicon-length range does not match with any number of repeats in the standard allele-calling table, the MIRU-profiler flags the locus as “UNKNOWN_ALLELE_NUM”. Under the default settings, the discrepancy range is set to ±5 bp to reduce the number of indeterminate loci without compromising the accuracy (Fig. S1).

The MIRU-profiler assumes that the source for the genome sequence is a pure culture from a single bacterial colony. Two alleles for the same locus (double alleles), which indicates mixed-culture or mixed-infection, are not reported by the MIRU-profiler. For detailed troubleshooting in case of “UNKNOWN_ALLELE_NUM” or “NOT_DETECTED”, MIRU-profiler generates an intermediate output file which describes the amplicon lengths for 24 MIRU-VNTR loci scanned in an input genome sequence.

### Bacterial strains for the validation

Four datasets were used to evaluate the accuracy of the MIRU-profiler results with respect to experimental genotyping and a web-based tool for digital MIRU-VNTR typing of *M. tuberculosis* (CASTB).

The sequencing and assembly details of all samples are summarized in Tables S2 and S3. The datasets were compiled through literature reviews (search terms described below). Publications in which the order of the 24-loci MIRU-VNTR in the numeric pattern for MIRU-VNTR profile was not clearly indicated were excluded. In addition, publications describing MIRU-VNTR results on non-standard set of VNTR loci were also not included for the validation.

#### Finished whole-genome-sequences from NCBI-GenBank

The first dataset comprised of 11 whole-genome-sequenced *M. tuberculosis* strains for which the experimental 24-loci MIRU-VNTR profiles have been described in previous publications (Clemmensen et al., 2017; Ilina et al., 2013; Kotłowski, 2015; Liao et al., 2014; Mokrousov, 2013; Rodríguez-Castillo et al., 2017; Roetzer et al., 2013; Roetzer et al., 2011). These 11 strains were identified through a literature review as follows: (1) Searching over 6,000 published articles in the Genome Announcements (an American society for microbiology journal that publishes short descriptions on microbial genomes in the public databases) with keywords “Mycobacterium tuberculosis” AND “MIRU-VNTR”. (2) Searching PubMed for all complete genomes with their “Strain ID” OR “Assembly accession” OR “Bio Project accession”, followed by reading corresponding publications for experimental MIRU-VNTR results.

The genome assemblies for these strains were downloaded from national-center-for-biotechnology-information (NCBI, https://www.ncbi.nlm.nih.gov/genome/genomes/166). The 11 strains were whole-genome-sequenced using a combination of ABI3730xl, Roche-454, Illumina-GAIIx, Illumina-MiSeq or PacBio RS and de-novo assembled into a single contig.

#### Genome assemblies based on Pacbio sequencing

In addition, the MIRU-profiler was also tested on four circulating strains from Hong Kong, for which we performed complete genome-sequencing and described in our recent publications (Leung et al., 2017; Rajwani et al., 2018). The genomes of these four strains were sequenced on the PacBio RS II platform with average read-length >10 Kb and de-novo assembled into a single contig. The genome sequences of these strains are available from NCBI-nucleotide accession-numbers: CP022578, CP022577, CP019613, CP019610.

#### Assembled genomes for BCG vaccine strains

In order to determine whether the MIRU-profiler could also be used to determine the MIRU-VNTR profiles from the animal-adapted strains of the *M. tuberculosis complex* (such as *M. bovis*), the MIRU-profiler was tested on genomes of vaccine strains BCG Pasteur 1173P2 and BCG Tokyo 172, for which the complete genome sequences were available from the NCBI-Genbank (accession numbers NC_008769 and NC_012207) and the experimental MIRU-VNTR typing results have been previously described (Al-Hajoj et al., 2014).

#### Genome assemblies based on Illumina MiSeq

The MIRU-profiler was also assessed on a dataset of 106 strains for which both raw-sequencing reads and experimental genotyping results were available from the previous publications. A total of five independent studies reporting data on 106 strains whole-genome-sequenced with a read length of at least 250 bp on the Illumina Miseq sequencer were included in this dataset (Clemmensen et al., 2017; Lee et al., 2015; Merker et al., 2015; Sekizuka et al., 2015; Walker et al., 2018). This dataset was compiled by a PubMed search with terms “Illumina Miseq AND MIRU-VNTR”. The publications describing raw sequencing data with reads shorter than 250 bp were excluded.

The raw sequencing reads were downloaded from the European-nucleotide-archive (ENA) database. The genomes were denovo assembled using the A5-miseq pipeline with the default parameters (Coil, Jospin & Darling, 2014). The assembled genome sequences were subsequently used as input for the MIRU-profiler.

### Identification of sequence quality requirements

The effect of average sequencing depth on the MIRU-profiler was determined by reducing the sequencing depth of a sample and denovo assembling the reads, followed by the digital MIRU-VNTR analysis using the MIRU-profiler. For this purpose, the reads were down sampled from a representative sample (ERR2120287), which was sequenced at 150× and all 24-loci were detected, to low-sequencing depths i.e., 5×, 10×, 15×, 20×, 25× and 30×.

In addition, other requirements for input genome assembly such as the maximum number of contigs and minimum value for the average sequence quality of the raw data was determined using the receiver operating characteristic (ROC) curve analysis.

### Experimental MIRU-VNTR typing

The experimental 24-loci MIRU-VNTR typing for four Hong Kong strains were prospectively performed in this study. The typing was performed using established procedure described previously (Supply et al., 2006).

### Percentage agreement

For the evaluation of MIRU-profiler, percentage agreement between experimental genotyping and MIRU-profiler results was selected as a simple indicator of accuracy. It was calculated as the number of identical results, divided by the total number of comparisons, multiplied by one-hundred.

### Computational performance

The ability of the MIRU-profiler to process multiple genomes was tested on all complete *M. tuberculosis* genomes available from the NCBI-database (*n* = 157) (https://www.ncbi.nlm.nih.gov/genome/genomes/166; accessed on 23/02/2018). The execution times for the MIRU-profiler was computed using the GNU time program on 64-bit Ubuntu 16.04 LTS installed on an Intel Core M-5Y10c CPU ©0.80 GHZ × 4 with 4 GB RAM.

## Results

### Evaluation on the dataset from NCBI-database

The results of the first dataset comprising of 11 genomes downloaded from the NCBI-database are summarized in Table 1. In this dataset, the MIRU-profiler successfully determined the 24-loci MIRU-VNTR profile without any warnings of “UNKNOWN_ ALLELE_NUM” or “NOT_DETECTED” for any locus across all 11 samples. The overall median and average of percentage agreement across 24-loci between MIRU-profiler and experimental results was 100% and 96.59%, respectively. A complete agreement was observed for 20 MIRU-VNTR loci and between one to four mismatches for the remaining 4-loci. The 4-loci with mismatches were Mtub29 (two mismatches), Mtub30 (one mismatch), Mtub39 (two mismatches) and QUB26 (four mismatches).

**Table 1:** Comparisons among the MIRU-profiler, CASTB and experimental results.

GenBank accession	MIRU-profiler result^a^	CASTB result^a^	Experimental result^a^	Mismatched locus^b^
Dataset 1: Genome assemblies downloaded from the NCBI database.				
GCA_000008585	2-4-3-2-5-5-3-3-2-3-2-3-4-2-5-1-5-3-2-3-3-6-3-2	2-4-3-2-5-5-3-3-2-3-2-3-4-2-5-1-5-3-2-3-3-6-3-2	2-4-3-2-5-5-3-3-2-3-2-3-4-2-5-1-5-3-2-3-3-6-3-2	None
GCA_000193185	2-4-4-2-3-3-3-5-2-6-4-4-4-2-5-1-7-3-3-5-3-7-2-3	2-4-4-2-3-3-3-5-2-6-4-4-4-2-5-1-7-3-3-5-3-7-2-3	2-4-4-2-3-3-3-5-2-6-4-4-4-2-5-1-7-3-3-5-3-7-2-3	None
GCA_000224435	1-3-2-2-5-4-2-3-2-1-2-4-1-2-5-1-5-3-3-2- **2**-6-2-2	1-3-2-2-5-4-2-3-2-1-2-4-1-2-5-1-5-3-3-2- **2**-6-2-2	1-3-2-2-5-4-2-3-2-1-2-4-1-2-5-1-5-3-3-2- **3**-6-2-2	Mtub39
GCA_000331445	2-2-3-2-2-4-3-3-2-6-3-4-4-2-2-1-5-3-3-3-4-7-3-2	2-2-3-2-2-4-3-3-2-6-3-4-4-2-2-1-5-3-3-3-4-7-3-2	2-2-3-2-2-4-3-3-2-6-3-4-4-2-2-1-5-3-3-3-4-7-3-2	None
GCA_000350205	2-2-3-2-3-4-3-3-1-4-3-2-4-2-3-1-5-3-3-3- **9**- **2**-3-2	2-2-3-2-3-4-3-3-1-4-3-2-4-2-3-1-5-3-3-3- **9**- **2**-3-2	2-2-3-2-3-4-3-3-1-4-3-2-4-2-3-1-5-3-3-3- **5**- **9**-3-2	Mtub39, QUB26
GCA_000737385	2-2-4-2-3-2-3-3-2-3-3-4-2-1-5-1-6-3-3-2-3- **3**-2-2	2-2-4-2-3-2-3-3-2-3-3-4-2-1-5-1-6-3-3-2-3- **3**-2-2	2-2-4-2-3-2-3-3-2-3-3-4-2-1-5-1-6-3-3-2-3- **6**-2-2	QUB26
GCA_000954155	2-2-4-2-1-3-3-5-2-9-4-4-4-2-5-1-7-1-3-4-3-8-2-3	2-2-4-2-1-3-3-5-2-9-4-4-4-2-5-1-7-1-3-4-3-8-2-3	2-2-4-2-1-3-3-5-2-9-4-4-4-2-5-1-7-1-3-4-3-8-2-3	None
GCA_002116755	2-2-4-2-1-3-3-5-2-8-4- **2**- **2**-2-5-1-7-1-3-4-3- **1**-2-3	2-2-4-2-1-3-3-5-2-8-4- **2**- **2**-2-5-1-7-1-3-4-3- **1**-2-3	2-2-4-2-1-3-3-5-2-8- **4**- **4**-4-2-5-1-7-1-3-4-3- **8**-2-3	Mtub29, Mtub30, QUB26
GCA_002116795	2-2-4-2-1-3-3-5-2-9-4- **2**-4-2-5-1-7-1-3-4-3-8-2-3	2-2-4-2-1-3-3-5-2-9-4- **2**-4-2-5-1-7-1-3-4-3-8-2-3	2-2-4-2-1-3-3-5-2-9- **4**-4-4-2-5-1-7-1-3-4-3-8-2-3	Mtub29
GCA_002116835	2-2-4-2-1-3-3-5-2-9-4-2-2-2-5-1-7-1-3-4-3- **1**-2-4	2-2-4-2-1-3-3-5-2-9-4-2-2-2-5-1-7-1-3-4-3- **1**-2-4	2-2-4-2-1-3-3-5-2-9-4-2-2-2-5-1-7-1-3-4-3- **8**-2-4	QUB26
GCA_002116855	2-2-4-2-1-3-3-5-2-9-4-2-2-2-5-1-7-1-3-4-3-1-2-3	2-2-4-2-1-3-3-5-2-9-4-2-2-2-5-1-7-1-3-4-3-1-2-3	2-2-4-2-1-3-3-5-2-9-4-2-2-2-5-1-7-1-3-4-3-1-2-3	None
Dataset 2: Complete genome sequences of four local strains isolated from Hong Kong.				
CP019613	2-4-4-2-3-3-3-2-2-5-4-4-4-2-5-1-6-3-3-5-3-7-2-3	2-4-4-2-3-3-3-2-2-5-4-4-4-2-5-1-6-3-3-5-3-7-2-3	2-4-4-2-3-3-3-2-2-5-4-4-4-2-5-1-6-3-3-5-3-7-2-3	None
CP019610	2-4-4-2-1-3-3-5-2-5-4-4-4-2-5-1-7-1-3-5-3-8-2-3	2-4-4-2-1-3-3-5-2-5-4-4-4-2-5-1-7-1-3-5-3-8-2-3	2-4-4-2-1-3-3-5-2-5-4-4-4-2-5-1-7-1-3-5-3-8-2-3	None
CP022577	2-2-4-2-2-3-3-5-2-5-4-4-4-2-5-1-7-3-3-4-5-8-2-3	2-2-4-2-2-3-3-5-2-5-4-4-4-2-5-1-7-3-3-4-5-8-2-3	2-2-4-2-2-3-3-5-2-5-4-4-4-2-5-1-7-3-3-4-5-8-2-3	None
CP022578	2-2-4-2-2-3-3-5-2-5-4-4-4-2-5-1-7-3-3-4-5-8-2-3	2-2-4-2-2-3-3-5-2-5-4-4-4-2-5-1-7-3-3-4-5-8-2-3	2-2-4-2-2-3-3-5-2-5-4-4-4-2-5-1-7-3-3-4-5-8-2-3	None
Dataset 3: Complete genome sequences for BCG-vaccine strains				
NC_008769	2-0-6-2s-2-2-3-1-2-3-5-2-2-5-4-2-5-3-3-3-2-5-0-2	2-0-6-2s-2-2-3-1-2-3-5-2-2-5-4-2-5-3-3-3-2-5-0-2	2-0-6-2s-2-2-3-1-2-3-5-2-2-5-4-2-5-3-3-3-2-5-0-2	None
NC_012207	2-0-5-3s-2-2-3-1-2-3-5-2-2-5-4-2-5-3-3-3-2-4-0-2	2-0-5-3s-2-2-3-1-2-3-5-2-2-5-4-2-5-3-3-3-2-4-0-2	2-0-5-3s-2-2-3-1-2-3-5-2-2-5-4-2-5-3-3-3-2-4-0-2	None

**Notes.**

a The number of repeats at the 24 MIRU-VNTR loci are presented in a 24-digit numeric pattern where each-digit represents the number of repeats at a particular locus according to the following order of the loci: MIRU02-Mtub04-ETRC-MIRU04-MIRU40-MIRU10-MIRU16-Mtub21-MIRU20-QUB11b-ETRA-Mtub29-Mtub30-ETRB-MIRU23-MIRU24-MIRU26-MIRU27-Mtub34-MIRU31-Mtub39-QUB26-QUB4156-MIRU39. The mismatched loci are bold and underlined .

b Mismatched-locus  = any locus that do not agree between the MIRU-profiler and experimental result.

The results of the two digital MIRU-typing tools (i.e., MIRU-profiler and CASTB) were identical for the entire dataset. Therefore, the discrepancies between the digital and experimental results could not be regarded as a defect in implementation of the MIRU-profiler but a general inconsistency between whole-genome-sequencing and experimental MIRU-VNTR typing results.

### Evaluation on complete genomes of locally isolated strains sequenced with longer reads

The detailed results for the evaluation of MIRU-profiler on four locally isolated strains sequenced using PacBio technology are presented in Table 1. For all four strains, no discrepancies were observed in any comparison, unlike the first dataset where minor discrepancies were observed. This further emphasizes the importance of a careful experimental genotyping and good quality genome assembly for accurate in-silico inferences of 24-loci MIRU-VNTR profile.

### Evaluation on complete genomes of BCG-vaccine strains

The MIRU-profiler successfully determined the complete 24 MIRU-VNTR profile for the two BCG-vaccine strains. The results were identical to the experimental genotyping results and those obtained from the CASTB server (Table 1).

### Evaluation on the genome assemblies based on the Illumina MiSeq data

**Figure 2:**
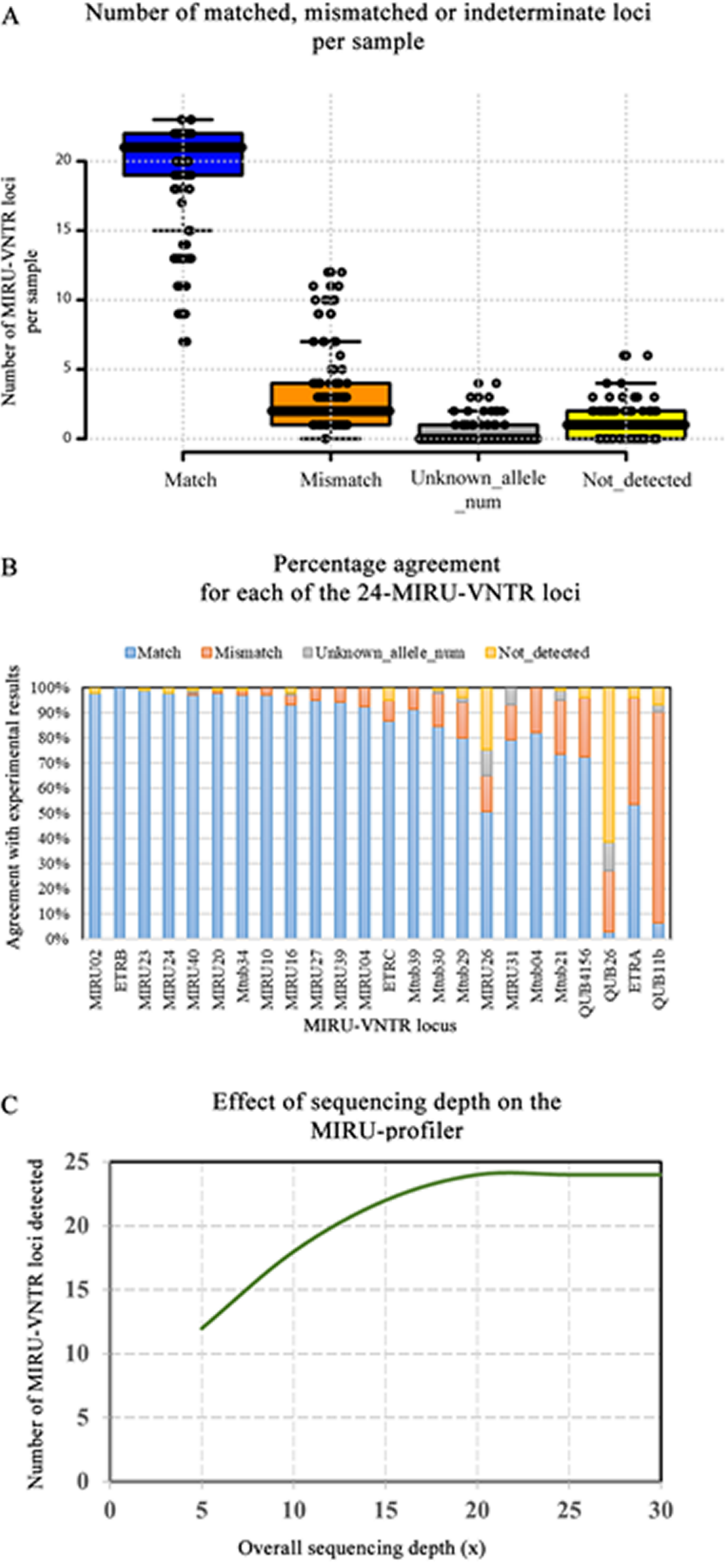
Evaluation of the MIRU-profiler on the genome assemblies based on Illumina Miseq reads. (A) A summary of the evaluation results is presented in the boxplot where number of matched, mismatched and indeterminate loci (unknown allele number or not detected) per sample are shown. (B) Percentage agreement between MIRU-profiler and experimental results for each locus is shown. (C) The effect of average sequencing depth on the number of loci that could be detected by the MIRU-profiler is shown. The analysis was performed by down sampling reads from one of the samples to 5×, 10×, 15×, 20×, 25× and 30×.

The results of the MIRU-profiler evaluation on the genome assemblies based on 250 bp paired-end reads from the Illumina MiSeq machine are detailed in Table S3 and illustrated in Fig. 2. All 24-MIRU-VNTR loci were detected in 29/106 (27.3%) samples. A single locus was not detected in 45/106 (42.4%), two loci were not detected in 22/106 (20.7%) and three or more loci were not detected in the remaining 10/106 (9.4%) samples. Among all 106 samples, one specific locus (QUB26) was not detected in 65/106 (61.3%) samples, which could be due the inability of the genome assembly algorithm to assemble this locus with relatively long repeat length (111 bp). In a majority (78/106; 73.5%) of the samples, none of the 24-loci was flagged as the unknown allele number by the MIRU-profiler.

In comparison with the experimental results, there were 2/106 (1.8%) samples with no mismatches, 30/106 (28.3%) with a single mismatch, 29/106 (27.3%) with two mismatches and 45/106 (42.4%) with three or more mismatches. Among the 30 samples with a single mismatch, one specific locus (QUB11b) was discrepant in 86.6% (26/30) of the samples. Similarly, among the samples with two mismatches, one of the two discrepant loci was QUB11b and the other being QUB26 (44.8%; 13/29), ETRA (20.6%; 6/29), QUB4156 (20.6%; 6/29), MIRU10 (6.89; 2/29), Mtub29 (3.4%; 1/29) or Mtub21 (3.4%; 1/29). The high number of discrepancies for the QUB11b locus could be attributed to both errors in the genome assembly and experimental genotyping. The samples with a greater number of mismatches (three or more) were associated with reduced quality of the raw sequencing data and a higher number of assembled contigs. Overall, the number of samples with a mismatch for any given locus was not greater than 26/106 (25%) except for the ETRA (45/106;42.7%) and QUB11b (89/106; 83.9%).

The accuracy of the MIRU-profiler is dependent on the quality of the input assembly, which further depends on the quality of the raw sequencing data. The effect of the average sequencing depth on the detection of the MIRU-VNTR is illustrated in Fig. 2. The increase in sequencing depth from 5× to 20× increases the number of MIRU-VNTR loci detected. The increasing trend plateaus at 20×, suggesting a minimum of 20× sequencing depth is sufficient to detect all 24 loci, while a further increase in depth to 30× or more do not have any effect on the performance of the MIRU-profiler. The MIRU-profiler was also observed to be affected by the sequence quality of the raw reads and the number of contigs in the final assembly. Based on a ROC curve analysis, the average of the phred-scale quality scores for the sequencing reads should be at least 37 and the final assembly should be assembled into no more than 179 contigs to obtain a reliable digital MIRU-VNTR result with at most three mismatches (Table S3).

Thus, the MIRU-profiler could accurately infer the digital MIRU-VNTR results with at most three mismatches from assemblies based on the Illumina MiSeq 250 bp paired-end reads, given the sequence quality requirements are fulfilled.

### Execution time for MIRU-profiler

The MIRU-profiler computed the result for the validation dataset of complete genomes in less than one minute (real 0m47.800s, user 0m43.496s and sys 0m1.096s). The computational time for processing a single file was less than five seconds (real 0m4.307s, user 0m4.012s and sys 0m0.120s), although with an extended run-time (real 14m49.848s, user 13m22.832s and sys 0m28.972s), the MIRU-profiler also executed successfully on a larger dataset of all published complete *M. tuberculosis* genomes (*n* = 157).

## Discussion

Here, we present the MIRU-profiler (a tool for digital MIRU-VNTR typing of *M. tuberculosis*) and evaluate its accuracy on four sets of experimentally genotyped and genome-sequenced strains, with widely different quality of genome assemblies. On all sets of *M. tuberculosis* strains, a good agreement between the MIRU-profiler and the experimental results was observed, which provides a confidence in in-silico inference of 24-loci MIRU-VNTR profiles from WGS using this tool. Other than *M. tuberculosis*, the MIRU-profiler also inferred accurate MIRU-VNTR profiles for the two BCG-vaccine strains, which indicates that the tool could possibly be applied to other members of the *Mycobacterium tuberculosis complex*, however, this must be further validated in the future.

The MIRU-profiler is the only available tool for digital MIRU-VNTR typing of multiple *M. tuberculosis* genomes, overcoming the limitations of existing tools for MIRU-VNTR analysis of *M. tuberculosis.* Previously available tools for MIRU-VNTR analysis do not offer an option to determine the 24-loci MIRU-VNTR profile from the genome sequence of *M. tuberculosis* or were limited in their throughput and accuracy. A recently developed web-based tool CASTB allows free service of digital MIRU-VNTR typing using assembled genome sequences (Iwai et al., 2015). However, the accuracy of the CASTB results was not compared with experimental results and it does not allow batch processing, which limits reliability and throughput of an analysis. In addition, unpublished genome sequences might need to be uploaded to the CASTB server, which raises further concerns about the data confidentiality. CASTB is also time inefficient. It requires uploading and downloading of data. Often, a request for analysis might need to be queued at the server-side before any results could be obtained. On the contrary, the MIRU-profiler results were compared to the experimental genotyping results in the current study. The MIRU-profiler is suitable for the bulk analysis of genome sequence data. It is more time efficient than the CASTB server, which is incapable of analyzing more than one genome at a time. The MIRU-profiler could be installed locally on any standard computer and the results could be obtained rapidly without any concerns on confidentiality.

Although the MIRU-profiler has several advantages such as the inference of digital MIRU-VNTR profiles from a large number of genomes in a single batch, some discrepancies were observed between the experimental and the digital MIRU-VNTR typing results. These discrepancies could be attributed to the errors in the interpretation of experimental results, microevolution or inaccuracies in the genome assembly. The experimental result is prone to errors due to sub-optimal PCR amplification, which results in multiple bands on an agarose gel electrophoresis and affects the interpretation of experimental results (Nikolayevskyy et al., 2016; Supply, 2005). The microevolution could also result in minor discrepancies between the experimental genotyping and WGS. Indeed, the most discrepant loci in our validation (QUB26 and QUB11b) were reported to be frequently associated with double alleles, a probable consequence of the microevolution (Jajou et al., 2017). The accuracy of the genome assembly, which crucially depends on the read length, is also vital to the digital inference of the MIRU-VNTR profile. The importance of a good quality genome assembly was quite evident from the validation results, where results nearly identical to the experimental genotyping were obtained for the complete genomes (e.g., locally isolated PacBio sequenced strains, BCG-vaccine strains and others downloaded from the NCBI-GenBank), while a few mismatches were observed for the genome assemblies based on Illumina MiSeq data. Nevertheless, even on the genome assemblies solely based on the Illumina MiSeq, at least 21/24 loci could be accurately inferred using the MIRU-profiler and the results for only three specific loci (QUB26, ETRA and QU11B) were unreliable due to the technological limitations. The current study validated the MIRU-profiler on the *M. tuberculosis* genomes assembled using reads 250 bp or longer, while a number of *M. tuberculosis* strains are currently also sequenced with reads 150 bp or shorter. The quality of the denovo assembly using relatively short reads (<150 bp) is rather low, particularly for the repetitive regions, therefore, genome assemblies based on reads 150 bp or shorter are inappropriate for the digital MIRU-VNTR analysis. The accuracy for the MIRU-profiler is best on the complete genomes assembled using long-read sequencing technologies (e.g., PacBio or Nanopore), with these technologies gradually becoming common in the clinical microbiology laboratories, the MIRU-profiler might be a useful solution to communicate with laboratories who may not have access to the WGS but perform MIRU-VNTR typing on a routine basis.

In conclusion, the MIRU-profiler is a rapid tool for the determination of MIRU-VNTR profiles from multiple assembled genome sequences of *M. tuberculosis*. The key features include fast computation, batch processing and data confidentiality. The accuracy of the MIRU-profiler is dependent on the quality of the input genome sequence. The results identical to the experimental typing could be obtained with high quality finished genomes. Thus, the MIRU-profiler might enable connections between the new genome sequenced isolates with a large number of unsequenced genotyped strains for epidemiological surveillance and research purposes.

##  Supplemental Information

10.7717/peerj.5090/supp-1 Table S1 Results for the MIRU-profiler evaluation on the draft genomes based on 150 bp reads

10.7717/peerj.5090/supp-2 Table S2 Details of complete genomes used for evaluation of the MIRU-profiler

10.7717/peerj.5090/supp-3 Table S3 Results for the MIRU-profiler evaluation on the draft genomes based on Illumina Miseq

10.7717/peerj.5090/supp-4 Figure S1 Optimal discrepancy range for the MIRU-profiler The effects of adjusting discrepancy range on the successful and accurate allele assignment by the MIRU-profiler is shown. The shown analysis was performed on the Illumina MiSeq dataset ( *n* = 106). The MIRU-profiler results were recorded by sequentially increasing the discrepancy range from 0 to 70 bp with a step of 5 bp. Increasing the discrepancy range to 5 bp increases the number of loci with a successfully determined repeat number by 1. Increasing the discrepancy range further has little advantage, while increasing discrepancy range to high numbers (such as 50) could significantly increase the chances of inaccurate results.

## Additional Information and Declarations

### Data Availability

The following information was supplied regarding data availability: Github: https://github.com/rahimrajwani/MIRU-profiler.
