# Supplementary figures and images for: MIRU-profiler: a rapid tool for determination of 24-loci MIRU-VNTR profiles from assembled genomes of Mycobacterium tuberculosis
Source: PeerJ. 2018 Jul 11;6:e5090. doi: 10.7717/peerj.5090 (PMC6045920; doi:10.7717/peerj.5090)

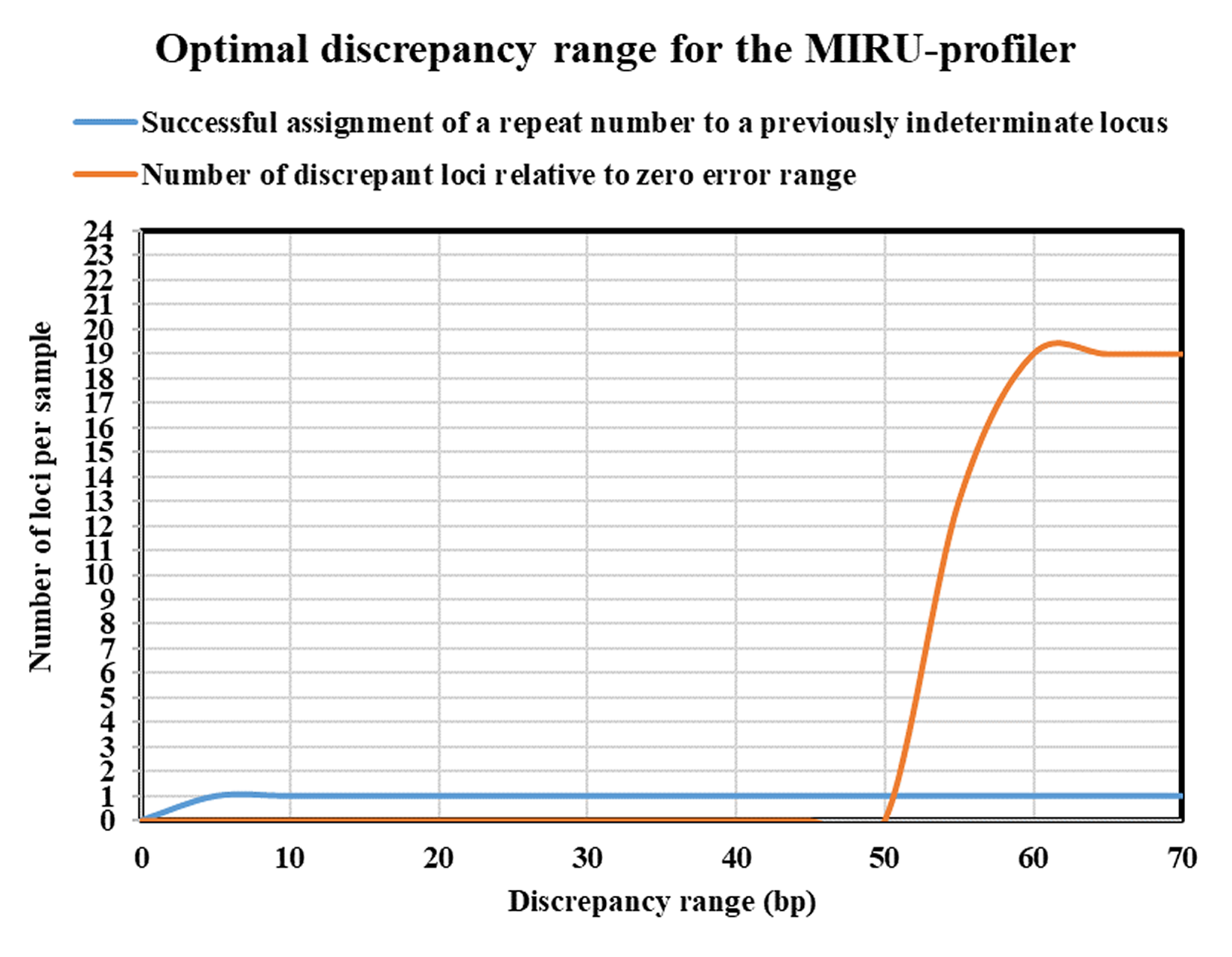

Supplement: Figure S1 — The effects of adjusting discrepancy range on the successful and accurate allele assignment by the MIRU-profiler is shown. The shown analysis was performed on the Illumina MiSeq dataset ( n = 106). The MIRU-profiler results were recorded by sequentially increasing the discrepancy range from 0 to 70 bp with a step of 5 bp. Increasing the discrepancy range to 5 bp increases the number of loci with a successfully determined repeat number by 1. Increasing the discrepancy range further has little advantage, while increasing discrepancy range to high numbers (such as 50) could significantly increase the chances of inaccurate results. [file peerj-06-5090-s004.png]
